# Dihydrojasmone from antifungal bacterial endophytes as a biocontrol agent against leaf spot pathogens threatening the endangered Tertiary relict plant *Parrotia subaequalis*

**DOI:** 10.3389/fpls.2025.1694888

**Published:** 2025-12-04

**Authors:** Mingmei Yang, Xingjian Liu, Wenhao Zhu, Zhanghua Quan, Jiahao Shen, Ren Wang, Jiayu Zhou

**Affiliations:** 1 Institute of Botany Jiangsu Province and Chinese Academy of Sciences, Nanjing, China; 2 Jiangsu Key Laboratory for Conservation and Utilization of Plant Resources, Nanjing, China; 3 Nanjing University of Chinese Medicine, Nanjing, China; 4 College of Biological Sciences and Technology, Yili Normal University, Yining, China

**Keywords:** *Parrotia subaequalis*, bacterial endophytes, fungal pathogens, biocontrol, dihydrojasmone, δ-tridecalactone

## Abstract

**Introduction:**

*Parrotia subaequalis*, an endangered Tertiary relict species endemic to eastern China, holds significant values for both evolutionary ecology and ornamental horticulture. However, it is increasingly threatened by leaf spot disease. The causal pathogens and the potential role of bacterial endophytes in disease suppression remain uncharacterized.

**Methods:**

In this study, fungal pathogens were isolated from diseased leaves collected from native habitats and their pathogenicity was confirmed by Koch’s postulates. Simultaneously, bacterial endophytes were isolated from the non-lesioned portions of diseased leaves and screened for their antagonistic activity. Additionally, the antifungal potential of bacterial secondary metabolites was evaluated and analyzed using untargeted metabolomic profiling.

**Results:**

Four fungal strains, *Alternaria* sp. S1, *Schizophyllum* sp. S2, *Diaporthe* sp. S5, and *Botryosphaeria* sp. S6, were confirmed as causal agents of leaf spot disease, with S5 causing the most severe symptoms. A total of 206 bacterial endophytes were isolated and 25 strains exhibited strong inhibition against fungal pathogens. Among them, *Bacillus* species showed the strongest antagonistic effects. Additionally, there were 103 bacterial metabolites identified. Seven abundant metabolites were tested for their antifungal activity, revealing that dihydrojasmone (DJ) and δ-tridecalactone (DT) significantly inhibited fungal growth. DJ displayed broad-spectrum inhibition, suppressed fungal melanin synthesis, and induced hyphal deformation, while DT exerted weaker but significant effects against S2 and S6.

**Discussion:**

This study provides the first identification of fungal pathogens responsible for leaf spot disease in *P. subaequalis*. The strong antifungal activities of endophytic Bacillus species and their metabolites were demonstrated, highlighting their promise as eco-friendly biocontrol agents.

## 1 Introduction

*Parrotia subaequalis* (Hamamelidaceae) is a Tertiary relict species endemic to China, with only eight known populations confined to subtropical mountainous regions across several eastern provinces, including Anhui, Jiangsu, Zhejiang, and Henan (Zhang et al., 2018). As a relict taxon, it serves as an important model for research in evolutionary ecology and conservation biology (Zhang et al., 2025). However, narrowly distributed species are generally more vulnerable to environmental fluctuations and anthropogenic disturbances (Qiu et al., 2023). In its native habitats, *P. subaequalis* is increasingly threatened by leaf spot and leaf blight diseases, which not only reduce its ornamental value by damaging its colorful leaves but also impair photosynthesis and consequently inhibit plant growth (Yang et al., 2024). These diseases are therefore regarded as potential drivers of its endangerment, contributing to its designation as a nationally first-level protected wild plant in China (Mao et al., 2024). Our field investigations confirmed that *P. subaequalis* individuals across six native habitats, including Xinyang (Henan Province), Anqing and Xuancheng (Anhui Province), Yixing (Jiangsu Province), and Ningbo and Huzhou (Zhejiang Province), all suffered from leaf spot disease to varying degrees (**Supplementary Table S1** and **Supplementary Figure S1**). Despite its widespread occurrence, the causal pathogens of leaf spot disease in *P. subaequalis* remain uncharacterized, and no effective biocontrol strategies are currently available.

Bacterial endophytes are microorganisms that reside within plant tissues without causing apparent damage (Hardoim et al., 2008). Increasing evidence suggests that plants can recruit bacterial endophytes with antagonistic abilities in response to pathogens. For example, infection by *Fusarium oxysporum* significantly altered the bacterial community composition in *Capsicum annuum* with enrichment of genera such as *Pseudomonas*, *Streptomyces*, *Klebsiella*, *Enterobacter*, *Microbacterium*, and *Bacillus* (Gao et al., 2021). Similarly, infections by *Magnaporthe grisea* and *Ustilaginoidea virens* induced distinct shifts in the bacterial communities of rice panicles, resulting in the enrichment of distinct genera (Wang et al., 2024a). Several bacterial endophytes, including *Pantoea agglomerans*, *Pantoea jilinensis*, *Acidovorax wautersii*, *Burkholderia contaminans*, and *Burkholderia pyrrocinia*, exhibited strong antagonistic effects against both pathogens. Furthermore, in sugar beet infected by *Rhizoctonia solani*, bacterial endophytes were found to activate gene clusters responsible for the biosynthesis of several antifungal metabolites, including phenazines, polyketides, and siderophores (Carrión et al., 2019). Collectively, pathogen challenge is often associated with compositional or functional changes in bacterial communities *in planta*, which can serve as reservoirs of biocontrol agents.

Bacterial endophytes can protect plants against pathogens via various mechanisms (Liu et al., 2022). Indirectly, they can enhance plant immunity and resistance to pathogens (Pieterse et al., 2014). More directly, they can produce a variety of bioactive metabolites with antifungal activity (Liu et al., 2017). For example, *Bacillus amyloliquefaciens* produces a series of iturins able to suppress *Verticillium dahliae* and protect cotton against Verticillium wilt (Han et al., 2015). It also synthesizes other lipopeptides, including surfactin and fengycin, which inhibit *Fusarium oxysporum* f. ap. *nivenum* and confer protection to watermelon against *Fusarium* wilt (Al-Mutar et al., 2023), while *Bacillus velezensis* releases several compounds, such as isobutyric acid, trans-2-octenal, tiglic acid, and 2-decanone, capable of inhibiting *Agroathelia rolfsii* and controlling southern blight of industrial hemp (Wang et al., 2024b). Additionally, *Pseudomonas fluorescens* could protect *Atractylodes lancea* from southern blight by secreting dimethyl disulfide and 2-piperidinone able to inhibit *Athelia rolfsii* (Zhou et al., 2014). Therefore, bacterial endophytes and their metabolites are increasingly recognized as environmentally sustainable biocontrol agents for plant disease management.

To date, the causal agents of leaf spot disease in *P. subaequalis* remain uncharacterized, posing a critical challenge for the conservation of this endangered Tertiary relict species. Bacterial endophytes, known for their antagonistic activities against plant pathogens, offer a promising strategy for sustainable disease management. This study aims to isolate bacterial endophytes from leaves of *P. subaequalis* across six native habitats and to identify strains with antagonistic activities against leaf spot pathogens. The findings are expected to provide novel insights into the interactions among plants, pathogens, and endophytes, providing strategies for protection and conservation of this rare species.

## 2 Materials and methods

### 2.1 Leaf sample collection and surface sterilization

In June 2023, leaf samples of *Parrotia subaequalis* were collected from six native habitats: Xinyang (XY), Anqing (AQ), Xuancheng (XC), Yixing (YX), Ningbo (NB), and Huzhou (HZ) (**Supplementary Table S1**). Following systematic sampling, three plots (5 m × 5 m) were established per site, with more than 10 m between each other. Two individuals of *P. subaequalis*, spaced approximately 5 m apart, were randomly selected in each plot, and a leaf-bearing branch (length = 20–40 cm) was collected from the lower crown from each tree. Thus, six branches were collected at each site, and one leaf showing leaf spot symptoms per branch was selected for pathogen and bacterial endophyte isolation. The leaves were surface sterilized by sequential immersion in 75% ethanol for 5 s, 5% sodium hypochlorite for 2 min, and 75% ethanol for 2 min, followed by three washes with sterilized water. Excess water was removed before subsequent pathogen and bacterial endophyte isolation.

### 2.2 Fungal pathogen isolation and identification

Since bacterial pathogens typically produce water-soaked lesions in leaves (Gao et al., 2014), which were not observed in *P. subaequalis* leaves (**Supplementary Figure S1**), we inferred that the leaf spot disease was likely caused by fungal pathogens. Because *P. subaequalis* plants in XY and XC exhibited the highest disease incidence (**Supplementary Table S1**), fungal isolation was conducted from diseased leaves collected from these two sites. Specifically, leaf segments from the junction between healthy and symptomatic tissues were excised, placed on potato dextrose agar (PDA) plates, and incubated at 28 °C for 7 days. Morphologically distinct colonies were purified by sub-culturing on fresh PDA plates, and single colonies were subsequently stored in 20% glycerol at -80 °C.

Fungal identification was performed using both morphological and molecular approaches (Zhou et al., 2020). For microscopic observation, 8-mm fungal discs were cultured on PDA at 28 °C for 2 days, after which a sterilized coverslip was inserted obliquely into the medium approximately 0.5 cm from the colony edge. Once fungal mycelia extended to cover about one-third of the coverslip, the mycelial structure was examined using an Eclipse 80i Upright Microscope (Nikon Corporation, Shinagawa, Tokyo, Japan). For molecular identification, fungal genomic DNA was extracted from the outermost mycelia using the E.Z.N.A.^®^ HP Fungal DNA Kit (Omega Bio-Tek, Inc., Norcross, GA, USA). The 18S rRNA gene was amplified with primers NS1 (5’-GTAGTCATATGCTTGTCTC-3’) and NS4 (5’-CTTCCGTCAATTCCTTTAAG-3’) (Tennakoon et al., 2022). The amplified products were purified and sequenced by Sangon Biotech Co., Ltd (Shanghai, China). The resulting ITS sequences were compared with those in GenBank using Basic Local Alignment Search Tool (BLAST) search.

### 2.3 Fungal pathogen inoculation and re-isolation

The pathogenicity of each fungal isolate was assessed by fulfilling Koch’s postulates to confirm its role as the causal agent of leaf spot disease in *P. subaequalis*. Healthy leaves were collected from Nanjing Botanical Garden Memorial Sun Yat-Sen (118°83’E; 32°05’N), Jiangsu Province, China. The detached leaves were washed three times with sterilized water and placed on sterilized filter paper in Petri dishes (diameter = 9 cm) containing 8 mL of sterilized water, which maintained a consistently moist environment for the leaves throughout the experiment. Fungal inoculation was conducted following the method described by Zamani-Noor and Jedryczka (2025) with some modifications. In detail, four cross-shaped incisions were made on each leaf using a sterilized knife, and fungal discs (diameter = 4 mm) were placed upside down onto the incisions. Mock leaves were inoculated with PDA discs. For each fungus, two healthy leaves were inoculated, and four fungal discs were placed on each leaf. All inoculated leaves were incubated in a growth chamber (22 °C; 10 h light/14 h dark; light density 100 μmol m^-2^ s^-1^). Disease symptoms were observed daily. After the appearance of characteristic leaf spots, the fungal pathogen was re-isolated from the junction between healthy and symptomatic tissues and cultured on PDA plates. The re-isolated isolates were identified morphologically and molecularly to confirm their identity with the original isolates.

### 2.4 Bacterial endophyte isolation and identification

After surface sterilization, the non-lesioned portions of six leaves from each site were ground with approximately 1 g of sterilized quartz sand in 10 mL sterilized water, and the resulting homogenate was serially diluted. Aliquots of 150 μL from each dilution were spread onto Luria-Bertani (LB) agar plates in quadruplicate. The plates were incubated at 28 °C for 3 days, after which single colonies were purified and stored in 20% glycerol at -80 °C. For identification, the 16S rRNA gene of all 206 isolates was amplified with primers 27F (5’-AGAGTTTGATCCTGGCTCAG-3’) and 1492R (5’-GGTTACCTTGTTACGACTT-3’) (Liu et al., 2020).

To further confirm the taxonomic assignments of the 25 strains selected for subsequent experiments, their *gyrB* genes were amplified with primers G3F (5’- GAAGTCATCATGACCGTTCTGCAYGCNGGNGGNAARTTYGA-3’) and G3R (5’-AGCAGGGTACGGATGTGCGAGCCRTCNACRTCNGCRTCNGTCAT-3’) (Li et al., 2023). The amplified products were purified and sequenced, and the resulting sequences were compared with those in GenBank using BLAST search.

### 2.5 Effects of bacterial endophytes on fungal pathogens

A total of 206 bacterial endophytes were isolated from the leaves of *P. subaequalis* from six native habitats and a phylogenetic tree was constructed based on their 16S rRNA sequences (**Supplementary Figure S2**). Considering both phylogeny and geographical origin, 74 strains were selected for preliminary *in vitro* screening of antagonistic activity against fungal pathogens. Bacterial isolates were recovered from -80 °C glycerol stocks by streaking onto LB agar plates and incubated at 28 °C for 2 days. Bacterial cells were collected from the plate and washed twice in 10 mM MgSO_4_, with centrifugation in between for 5 min at 4,500 *g*. The bacterial cells were then suspended in 5 mL of 10 mM MgSO_4_ (Verbon et al., 2023).

The *in vitro* antifungal assays were carried out following the dual culture method described by Castaldi et al. (2021) with some modifications (**Supplementary Figure S3**). Fungal discs (diameter = 8 mm) were taken from the outmost edge of fungal colonies and placed upside down at the center of fresh LB plates. Three drops (10 μL each) of individual bacterial suspension and one drop of 10 mM MgSO_4_ were spotted equidistantly around the edge of the fungal colony. In parallel, a fungal colony treated with four drops of 10 mM MgSO_4_ in the same pattern was used as mock. Because this was a preliminary screening, the effect of each bacterial endophyte on different fungal pathogens was tested only once. All plates were incubated at 28 °C for 7 days, after which fungal colony diameters were measured using a cross-line method. The inhibition rate (IR) was calculated as follows:


$$\text{IR}\%=\frac{\left(\text{Mock colony dimeter}-\text{Treatment colony diameter}\right)}{\left(\text{Mock colony diameter}-\text{Fungal disc diameter}\right)}\times 100\%$$

If fungal growth was inhibited only in regions adjacent to the bacterial suspension but not near the MgSO_4_ spot, inhibition was primarily caused by soluble bacterial metabolites. If fungal growth was suppressed both near the bacterial suspension and the MgSO_4_ spot, compared with the mock, volatile bacterial metabolites were also considered to contribute to fungal inhibition. Bacterial strains exhibiting an IR greater than 70% against at least one fungal pathogen were selected. Again, their inhibitory activity was confirmed in four independent experiments using the same procedure described above.

### 2.6 Antifungal activity of bacterial fermentation supernatants

There were 25 bacterial strains showing IRs greater than 70% against at least one fungal pathogen; thus, their antifungal activity was repeatedly verified. Then, each strain was cultured in flask at 28 °C with agitation at 200 rpm for 3 days. Bacterial cells were removed by filtration through a 0.22-μm sterile filter to obtain the cell-free fermentation supernatant (Wang et al., 2024b). The filtered supernatant was mixed with LB agar (v/v = 1:4) and poured into Petri dishes. The discs (diameter = 8 mm) of each fungal pathogen were then inoculated upside down at the center of the plate. Each treatment was performed in quadruplicate, and the plates were incubated at 28 °C for 7 days. Fungal colony diameters were then measured, and the IR was calculated. The mock consisted of LB agar without the addition of bacterial fermentation supernatant.

### 2.7 Liquid chromatograph-mass spectrometer analysis of bacterial fermentation supernatants

For liquid chromatograph-mass spectrometer (LC-MS) analysis, 1.0 mL of filtered bacterial fermentation supernatant was transferred to a centrifuge tube, and 2 mL of extraction solvent (methanol/acetonitrile, 1:1, v/v) was added. The mixture was vortexed for 60 s and sonicated at 4 °C for 30 min. After centrifugation at 12,000 rpm for 10 min at 4 °C, the supernatant was collected and incubated at -20 °C for 1 h to precipitate proteins. The sample was centrifuged again at 12,000 rpm for 10 min, and the resulting supernatant was vacuum-dried. The residue was reconstituted in 100 μL of 30% acetonitrile, vortexed, and centrifuged at 12,000 rpm for 10 min at 4 °C. The final supernatant was analyzed using a UPLC system (Vanquish, Thermo Fisher Scientific, Waltham, MA, USA) coupled to a Q Exactive HFX Hybrid Quadrupole-Orbitrap mass spectrometer with a heated electrospray ionization (ESI) source in Full-ms-ddMS2 acquisition mode. The UPLC conditions were as follows: column, Waters HSS T3 (100 mm × 2.1 mm, 1.8 μm); mobile phases, A = ultrapure water with 0.1% formic acid and B = acetonitrile with 0.1% formic acid; flow rate, 0.3 mL/min; column temperature, 40 °C; injection volume, 2 μL. The gradient elution program was: 0–1 min; 0% B; 1–12 min, 0%-95% B; 12–13 min; 95% B; 13-13.1 min; 95%-0% B; and 13.1–17 min; 0% B. The ESI source parameters were as follows: spray voltage, -2.8 kV/+3.0 kV; sheath gas, 40 arb; auxiliary gas, 10 arb; sweep gas, 0 arb; capillary temperature, 320 °C; and auxiliary gas heater temperature, 350 °C. The MS scan range was 70–1050 Da, with a resolution of 70,000 (MS^1^) and 17,500 (MS^2^).

### 2.8 Antifungal activity of individual and combined bacterial metabolites

To identify specific antifungal compounds in the bacterial fermentation supernatants, seven candidate metabolites were selected for further validation based on the LC-MS results, including dihydrojasmone (DJ) (Adamas^®^, Shanghai Titan Scientific Co., Ltd., China), *p*-methylhippuric acid (PMA) (Adamas^®^, Shanghai Titan Scientific Co., Ltd., China), 4-aminobenzamide (4-AB) (Adamas^®^, Shanghai Titan Scientific Co., Ltd., China), phenylglyoxylic acid (PA) (Bide Pharmatech, Shanghai, China), δ-tridecalactone (DT) (Energy Chemical, Shanghai, China), *N*-deschlorobenzoyl indomethacin (NDI) (Bide Pharmatech, Shanghai, China), and (+)-bicuculline (BL) (Bide Pharmatech, Shanghai, China). The selection criteria were whether their structural analogues had been reported to exhibit antifungal activity, while compounds already well characterized as antifungal agents were deliberately excluded. In addition, the seven compounds were relatively abundant in the bacterial fermentation supernatants and were readily obtainable from commercial sources. Each compound was completely dissolved in dimethyl sulfoxide (DMSO) to prepare stock solutions at a concentration of 100 mg/mL and sterilized by filtration through a 0.22-μm sterile filter. A 200 μL aliquot of each stock solution was added to 100 mL of LB agar preheated to 50 °C, thoroughly mixed, and poured into Petri dishes (diameter = 9 cm; 12.5 mL per dish). The final concentration of each compound in the LB agar was 200 mg/L. Fungal discs (diameter = 8 mm) were then inoculated upside down at the center of each plate. Each treatment was performed in quadruplicate, and the plates were incubated at 28 °C for 7 days, after which the IR was calculated. The mock consisted of LB agar supplemented with 0.2% DMSO.

Based on the inhibitory effects of the tested compounds against different fungal pathogens, the effects of DJ and DT on the mycelial structures of all fungal pathogens were assessed. Fungal discs were inoculated onto LB agar containing 200 mg/L DJ or DT and incubated at 28 °C for 10 days, after which the mycelial structures at the very edge of the colonies were directly examined under an Eclipse 80i Upright Microscope. Furthermore, DJ and DT were selected for further dose-response assay. LB agar plates were prepared containing each compound at concentrations of 0, 50, 100, 150, 200, and 300 mg/L, and fungal discs (diameter = 4 mm) were inoculated upside down at the center of the plates. After incubation at 28 °C for 4 days, colony diameters were measured to determine the EC50 values. Each concentration of DJ and DT included four replicates. Furthermore, the combinatorial effect of DJ and DT was assessed. The two compounds were mixed at 11 different ratios (0:10. 1:9, 2:8, 3:7, 4:6, 5:5, 6:4, 7:3, 8:2, 9:1, and 10:0), and each mixture was incorporated into LB agar at a total compound concentration of 200 mg/L. Fungal discs (diameter = 4 mm) were inoculated and incubated at 28 °C for 4 days, after which colony diameters were measured. Each ratio included five replicates.

### 2.9 Statistical analysis

Phylogenetic trees were constructed and visualized using MEGA version 11.0.13 and ITOL (https://itol.embl.de/). EC50 values were calculated with GraphPad Prism. All other data analyses were conducted in R version 4.1.3. The community composition and diversity of bacterial endophytes were analyzed at the genus level. Bacterial community composition was shown using stacked bar plots and bacterial diversity was assessed using the vegan package and shown using box plots. Stacked bar plots and box plots were generated with the ggplot2 package. UpSet plots were created with the UpSetR package. Heatmaps were generated with the pheatmap package. Partial least squares-discriminant analysis (PLS-DA) was performed using mixOmics package. One-way ANOVA was conducted using the agricolae package.

For inhibitory assays, results are presented as the median with the 25^th^ and 75^th^ percentiles. Comparisons of fungal colony diameters between the mock and individual compound treatments were performed using Student’s *t*-test. Comparisons among bacterial endophytes, different fermentation supernatants, or compound combinations were performed using One-Way ANOVA, followed by the least significant difference (LSD) test (*P* < 0.05).

## 3 Results

### 3.1 Fungal pathogens causing *Parrotia subaequalis* leaf spot disease

**Figure 1:**
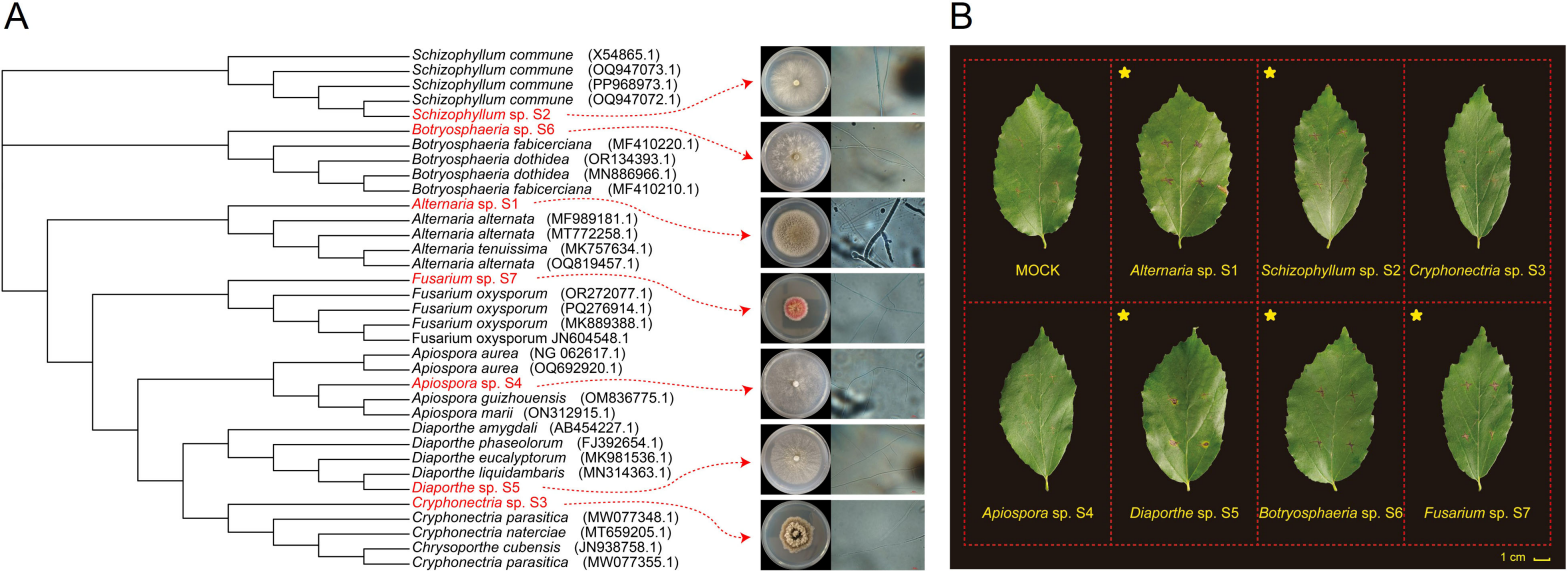
Identification and pathogenicity assays of potential fungal pathogens causing leaf spot disease in *Parrotia subaequalis*. **(A)** Phylogenetic tree and colony/microscopic characteristics of potential fungal pathogens. **(B)** Leaf spot symptoms on leaves inoculated with potential fungal pathogens. Yellow stars indicate that lesions could be caused by corresponding fungal strains. Leaves inoculated with potato dextrose agar (PDA) discs (MOCK) show no visible symptoms.

During field sampling, varying degrees of leaf spot disease were observed in *P. subaequalis* across six native habitats, with the highest incidence recorded in XY and XC (**Supplementary Table S1** and **Supplementary Figure S1**). Seven fungal strains were isolated and purified from the junction between healthy and symptomatic tissues. Morphological and molecular analyses identified these strains as *Alternaria alternata* (Aloi et al., 2021), *Schizophyllum commune* (Matsumae et al., 2025), *Cryphonectria parasitica* (Double et al., 2018), *Apiospora marii* (Kwon et al., 2022), *Diaporthe liquidambaris* (Gao et al., 2017a), *Botryosphaeria dothidea* (Zimowska et al., 2020), and *Fusarium oxysporum* (Kour et al., 2008). Thus, the seven strains were named *Alternaria* sp. S1, *Schizophyllum* sp. S2, *Cryphonectria* sp. S3, *Apiospora* sp. S4, *Diaporthe* sp. S5, *Botryosphaeria* sp. S6, and *Fusarium* sp. S7, respectively (**Figure 1A** and **Supplementary Table S2**). Among these fungal strains, S1-S5 were isolated from diseased leaves collected in XY and S6-S7 were isolated from those collected in XC.

To assess their pathogenicity, fungal discs were inoculated onto healthy leaves. Brown necrotic lesions developed on leaves inoculated with *Alternaria* sp. S1, *Schizophyllum* sp. S2, *Diaporthe* sp. S5, *Botryosphaeria* sp. S6, and *Fusarium* sp. S7 (**Figure 1B**). Among them, S5 caused the most severe symptoms, characterized by outwardly expanding lesions and yellowing of the surrounding tissues. Subsequently, fungi were re-isolated from the lesions, identified, and compared with the original isolates to fulfill Koch’s postulates. Except for S7, all inoculated fungal strains were successfully re-isolated (**Supplementary Figure S4**), confirming that S1, S2, S5, and S6 are the causal agents of leaf spot disease in *P. subaequalis*. The relative severity of damage was ranked as S5 > S1 > S6 > S2. Notably, S1, S2, and S5 were isolated from XY, while S6 was isolated from XC. These results suggest that not all pathogens infect *P. subaequalis* leaves simultaneously; instead, different pathogens may predominate at different habitats and give rise to similar leaf spot symptoms (**Figure 1B**). It is necessary to elucidate the dynamics of pathogen occurrence under field conditions in future studies.

### 3.2 Culturable bacterial communities in the leaves of *P. subaequalis* across six habitats

**Figure 2:**
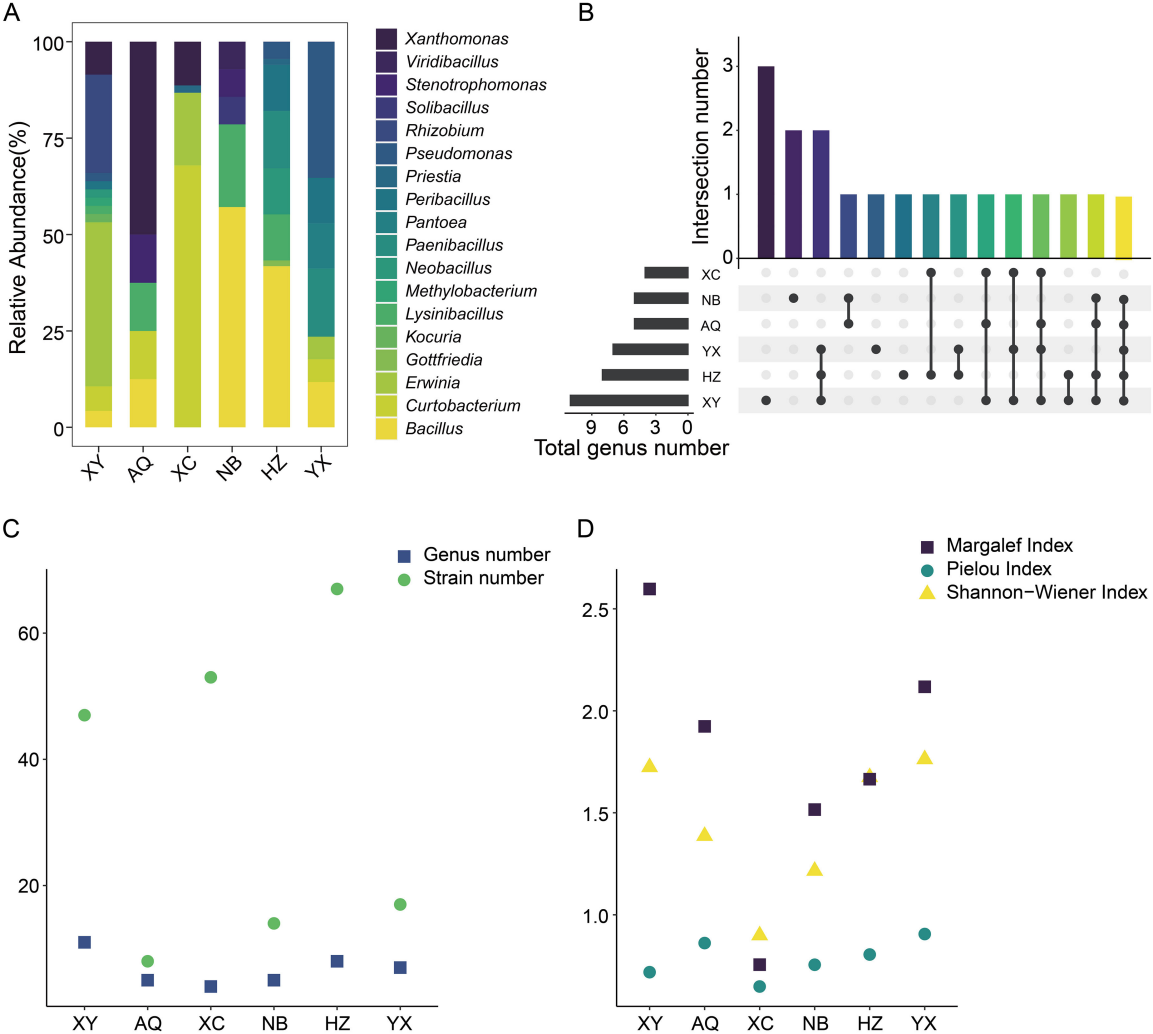
Community composition and diversity of culturable bacterial endophytes in leaves of *Parrotia subaequalis* across six native habitats. **(A)** Relative abundance of bacterial endophytes at genus level. **(B)** Genera shared among habitats or unique to each habitat. **(C, D)** α-diversity of bacterial endophytes across habitats. XY: Xinyang (Henan Province); AQ: Anqing (Anhui Province); XC: Xuancheng (Anhui Province); NB: Ningbo (Zhejiang Province); HZ: Huzhou (Zhejiang Province); YX: Yixing (Jiangsu Province).

A total of 206 culturable bacterial endophytes were isolated from the non-lesioned portions of diseased leaves of *P. subaequalis* collected from six native habitats (**Supplementary Table S3**). These bacterial endophytes were identified to 18 genera (**Supplementary Figure S2**). The bacterial community composition varied significantly across habitats (**Figure 2A**). In NB and HZ, *Bacillus* was the most abundant genus, representing 57.14% and 41.79% of the isolates, respectively. *Erwinia* was the dominant genus in XY (42.55%), *Xanthomonas* in AQ (50%), *Curtobacterium* in XC (67.92%), and *Pseudomonas* in YX (35.29%). Under our experimental conditions, no bacterial genus was shared across all habitats (**Figure 2B**), indicating habitat-specific differences in bacterial assemblages. Additionally, the α-diversity of bacterial endophytes differed significantly among habitats. Although the number of bacterial endophytes isolated from leaves collected in XY was not the highest, this habitat harbored the greatest number of bacterial genera (**Figure 2C**), consistent with its high Margalef and Shannon-Wiener indexes (**Figure 2D**). Notably, leaf spot disease was most severe in XY (**Supplementary Table S1** and **Supplementary Figure S1**), which may have facilitated the recruitment of diverse bacteria colonizing *P. subaequalis* leaves. In contrast, HZ harbored the largest number of bacterial strains, but its Margalef, Pielou, and Shannon-Wiener indexes were not the highest among all habitats. In future studies, additional approaches such as amplicon sequencing could help further reveal the bacterial composition and diversity in *P. subaequalis* leaves across habitats.

### 3.3 Inhibitory effects of bacterial endophytes on fungal pathogen growth

**Figure 3:**
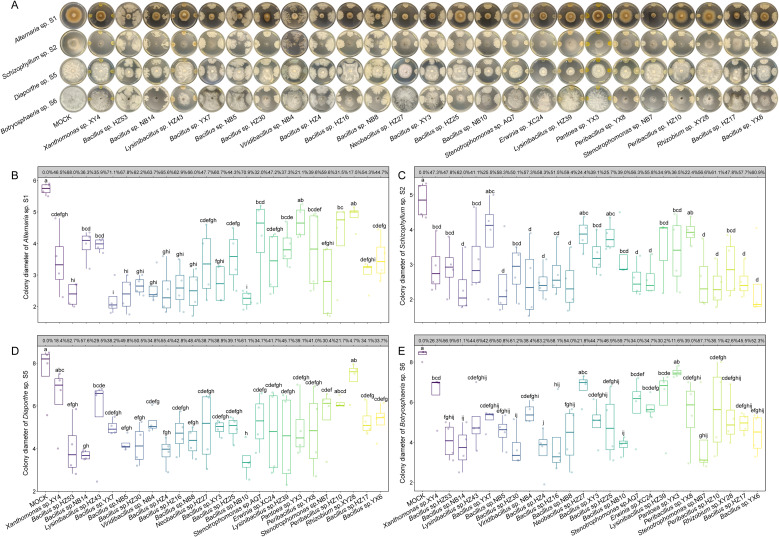
Inhibitory effects of bacterial endophytes on the growth of fungal pathogens. **(A)** Representative images of *in vitro* antagonistic assays. **(B-E)** Boxplots of colony diameters of *Alternaria* sp. S1 **(B)**, *Schizophyllum* sp. S2 **(C)**, *Diaporthe* sp. S5 **(D)**, and *Botryosphaeria* sp. S6 **(E)** treated by 10 mM MgSO_4_ or different bacterial suspensions. The inhibition rates for each bacterial endophyte are displayed in the grey boxes above the boxplots. The horizontal line inside each box represents the median; the top and bottom edges of each box represent the 75^th^ and 25^th^ quartiles, respectively; and the upper and lower whiskers extend to 1.5× the interquartile range from the top and bottom of the box, respectively. Different letters represent significant differences among suspensions of indicated bacterial endophytes (One-way ANOVA with least significant difference test, *P* < 0.05, *N* = 4).

A total of 74 bacterial endophytes were selected for preliminary antifungal screening, representing both phylogenetic diversity and geographic distribution (**Supplementary Figure S5**). Most strains, particularly those belonging to *Bacillus*, exhibited obvious inhibitory effects, with 25 strains showing IRs greater than 70% against at least one fungal pathogen (**Supplementary Figure S5**). The taxonomic assignments of these 25 antifungal strains based on *gyrB* genes (**Supplementary Table S4**) were consistent with those based on 16S rRNA sequences (**Supplementary Table S3**), supporting the robustness of bacterial identification. Subsequent repeated assays confirmed that most strains consistently suppressed fungal growth (**Figure 3**). Among them, *Bacillus* sp. YX7 exhibited the strongest inhibition against *Alternaria* sp. S1 (71.06%), *Bacillus* sp. YX6 against *Schizophyllum* sp. S2 (60.89%), *Bacillus* sp. NB10 against *Diaporthe* sp. S5 (61.13%), and *Bacillus* sp. HZ4 against *Botryosphaeria* sp. S6 (63.20%).

It is noteworthy that the incidence of leaf spot disease in each region appeared to be related to the number of antifungal bacterial endophytes isolated. Specifically, leaf samples from HZ exhibited the lowest disease incidence, and 10 out of the 25 bacterial endophytes with antifungal activity were isolated from this location (**Figure 3**). Among them, *Bacillus* sp. HZ4 and *Bacillus* sp. HZ16 showed remarkable antifungal activity. Furthermore, considering the broad-spectrum antifungal performance against four pathogens, *Bacillus* sp. YX6 isolated from YX was the most effective strain, while YX was another region with relatively low disease incidence.

### 3.4 Inhibitory effects of bacterial fermentation supernatant on fungal pathogen growth

**Figure 4:**
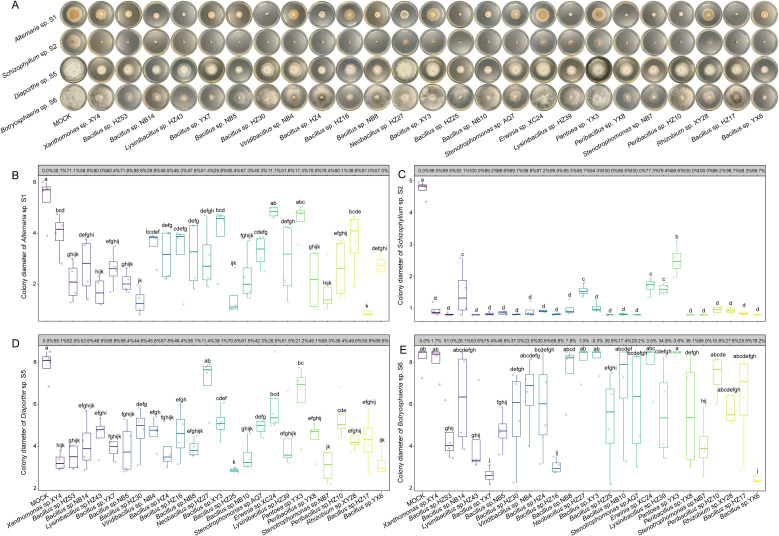
Inhibitory effects of bacterial fermentation supernatants on the growth of fungal pathogens. **(A)** Representative images of *in vitro* antagonistic assays. **(B-E)** Boxplots of colony diameters of *Alternaria* sp. S1 **(B)**, *Schizophyllum* sp. S2 **(C)**, *Diaporthe* sp. S5 **(D)**, and *Botryosphaeria* sp. S6 **(E)** treated by 10 mM MgSO_4_ or different bacterial fermentation supernatants. The inhibition rates for each bacterial fermentation are displayed in the grey boxes above the boxplots. The horizontal line inside each box represents the median; the top and bottom edges of each box represent the 75^th^ and 25^th^ quartiles, respectively; and the upper and lower whiskers extend to 1.5× the interquartile range from the top and bottom of the box, respectively. Different letters represent significant differences among supernatants of indicated bacterial endophytes (One-way ANOVA with least significant difference test, *P* < 0.05, *N* = 4).

As shown in **Figure 3**, most fungal colonies exhibited substantially different growth rates between the 10 mM MgSO_4_ side and the bacterial suspension sides, indicating that soluble antifungal metabolites secreted by bacterial endophytes play a more dominant role in inhibiting fungal growth than the uniformly distributed volatile metabolites in the Petri dishes (**Supplementary Figure S3**). Therefore, we assessed the inhibitory effects of bacterial fermentation supernatants on fungal pathogen growth (**Figure 4A**). The fermentation supernatants of 15 bacterial strains significantly suppressed the growth of *Alternaria* sp. S1, with IRs exceeding 50% (**Figure 4B**). Except for *Pantoea* sp. YX3, the supernatants of the remaining 24 strains significantly inhibited *Schizophyllum* sp. S2 (IR > 70%) (**Figure 4C**). The supernatants of 13 strains significantly inhibited *Diaporthe* sp. S5 (IR > 50%) (**Figure 4D**). However, the supernatants of only 6 strains showed significant inhibitory activity against *Botryosphaeria* sp. S6 (IR >50%) (**Figure 4E**). Collectively, these results indicate that bacterial endophytes can synthesize and secrete antifungal metabolites able to inhibit fungal growth, highlighting their antifungal potential at the metabolic level.

Furthermore, differences were observed between the inhibitory effects of bacterial endophytes and their fermentation supernatants. For instance, *Bacillus* sp. YX7 exhibited the strongest inhibition against *Alternaria* sp. S1 (IR = 71.06%), whereas its fermentation supernatant was less effective (IR = 60.36%). Similarly, *Bacillus* sp. HZ4 displayed the strongest inhibitory effect against *Botryosphaeria* sp. S6 (IR = 63.20%), but its fermentation supernatant exhibited weaker inhibition (IR = 30.63%). In contrast, the fermentation supernatant of *Bacillus* sp. HZ17 was much more effective than the bacterium itself (IR = 54.31%), almost completely suppressing S1 (IR = 96.96%). Collectively, these findings highlight the complexity of antifungal mechanisms employed by bacterial endophytes and underscore the necessity of further identifying bioactive metabolites from bacterial fermentation supernatants.

### 3.5 Metabolite profiling of bacterial fermentation supernatants

**Figure 5:**
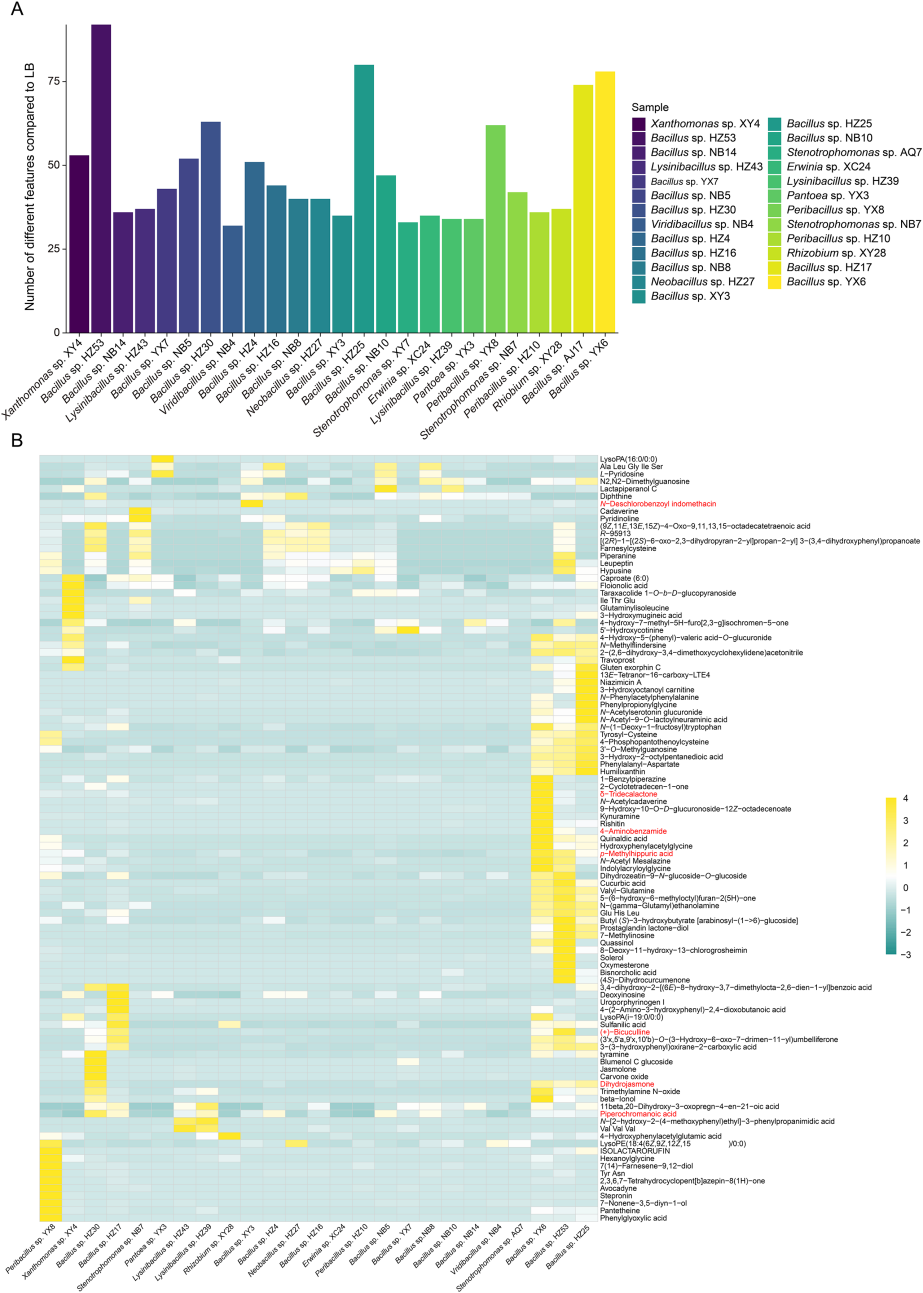
Untargeted metabolomic analysis of bacterial fermentation supernatants. **(A)** Number of compounds detected exclusively in bacterial fermentation supernatants compared to Luria-Bertani (LB) medium. **(B)** Heatmap of annotated compounds exclusively present in bacterial fermentation supernatants, with lower intensities shown in cyan and higher intensities in yellow. Compounds selected for further antifungal assays are highlighted in red.

Untargeted metabolomic analysis was conducted to characterize the metabolites present in the fermentation supernatants of the 25 bacterial endophytes screened above (**Supplementary Table S5**). PLS-DA revealed clear differences in metabolite composition among bacterial genera, with samples from *Bacillus* species clearly separated from others (**Supplementary Figure S6**). Notably, *Xanthomonas* sp. XY4 was distinctly separated from others along both Axis 1 and Axis 2. In total, 103 metabolites absent from LB liquid were identified as bacterial metabolites. Among the tested strains, the fermentation supernatant of *Bacillus* sp. HZ53 contained the highest number of metabolites, with a total of 92 identified (**Figure 5A**). However, only 32 metabolites were detected in *Viridibacillus* sp. NB4, representing the lowest among all strains. Overall, *Bacillus* strains produced more unique metabolites than other genera. Additionally, metabolite concentrations varied significantly among strains (**Figure 5B**). Based on these results, seven abundant and commercially available metabolites were selected for further assays, including dihydrojasmone (DJ), *p*-methylhippuric acid (PMA), 4-aminobenzamide (4-AB), phenylglyoxylic acid (PA), δ-tridecalactone (DT), *N*-deschlorobenzoyl indomethacin (NDI), and (+)-bicuculline (BL).

### 3.6 Effects of dihydrojasmone and δ-tridecalactone on fungal growth and mycelial morphologyar

**Figure 6:**
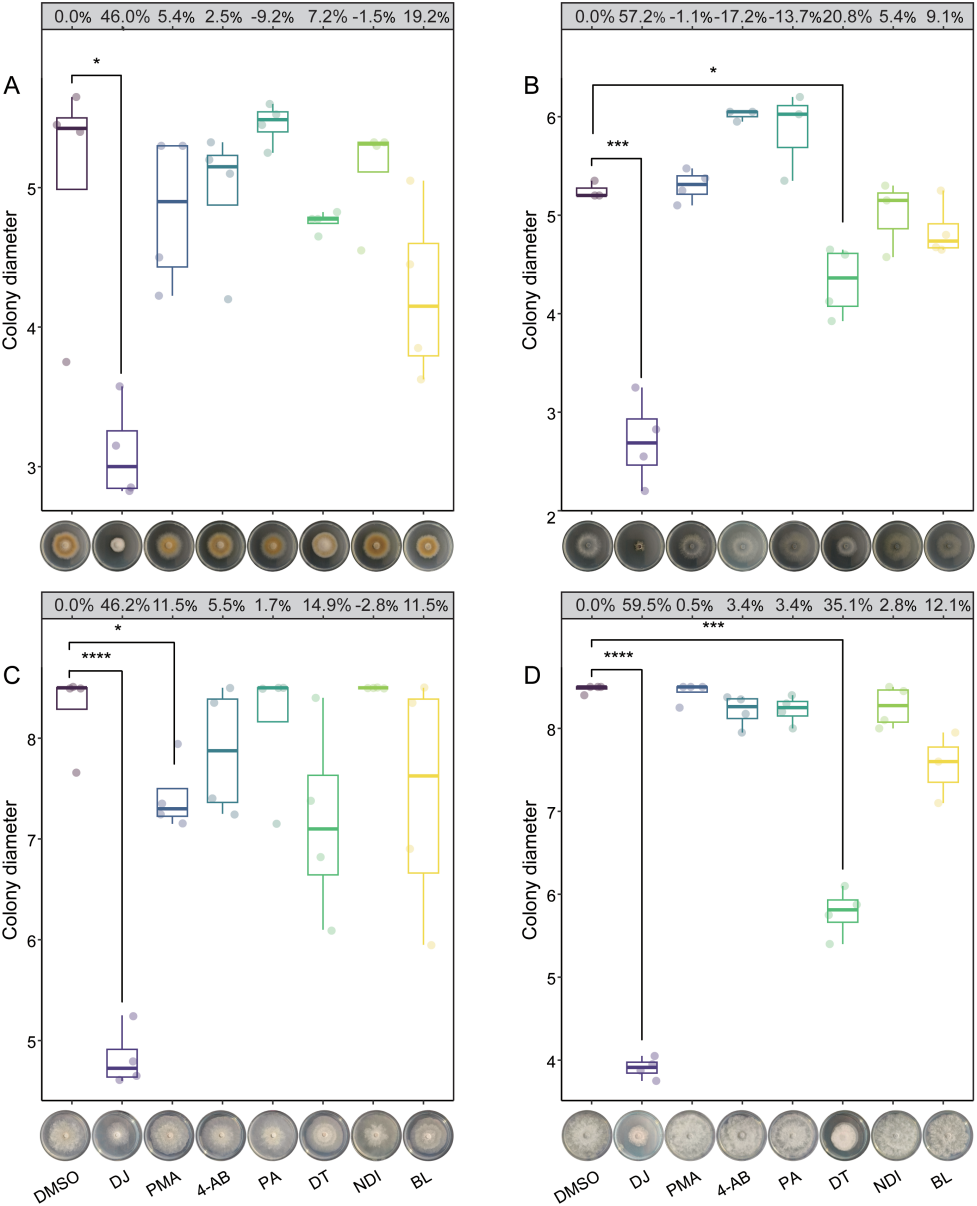
Effects of dihydrojasmone (DJ), *p*-methylhippuric acid (PMA), 4-aminobenzamide (4-AB), phenylglyoxylic acid (PA), δ-tridecalactone (DT), N-deschlorobenzoyl indomethacin (NDI), and (+)-bicuculline (BL) on the growth of *Alternaria* sp. S1 **(A)**, *Schizophyllum* sp. S2 **(B)**, *Diaporthe* sp. S5 **(C)**, and *Botryosphaeria* sp. S6 **(D)**. The inhibition rates for each compound are displayed in the grey boxes above the boxplots. The horizontal line inside each box represents the median; the top and bottom edges of each box represent the 75^th^ and 25^th^ quartiles, respectively; and the upper and lower whiskers extend to 1.5× the interquartile range from the top and bottom of the box, respectively. Asterisks represent statistically significant differences between dimethyl sulfoxide (DMSO) and each compound treatment (Student’s *t*-test; * *P* < 0.05, *** *P* < 0.001, **** *P* < 0.0001; *N* = 4).

The antifungal activities of DJ, PMA, 4-AB, PA, DT, NDI, and BL were evaluated against the four fungal pathogens. At the concentration of 200 mg/L, DJ significantly inhibited the growth of all the tested pathogens; DT significantly inhibited *Schizophyllum* sp. S2 and *Botryosphaeria* sp. S6; and PMA significantly inhibited *Diaporthe* sp. S5 (**Figure 6**). No significant inhibition was observed for the remaining compounds. Besides growth suppression, noticeable phenotypic changes were observed. Colonies of S1, which were brown under mock conditions, turned white after DJ treatment (**Figure 6A**). Additionally, all fungal colonies treated with DT exhibited denser and smoother edges compared to the mock. To further investigate these effects, the mycelial structures of all the fungal pathogens treated with DJ and DT were examined microscopically. As shown in **Supplementary Figure S7**, DT treatment induced varying degrees of wrinkling in the mycelia of S1 and S2, with more pronounced changes in S2. DJ treatment caused wrinkling and twisting specifically in the mycelia of S1. No obvious mycelial abnormality was obvious in S5 and S6.

Given their strong inhibitory activities, the EC50 values of DJ and DT were determined (**Supplementary Figures S8** and **S9**). The EC50 values of DJ against S1, S2, S5, and S6 were 115.4, 109.1, 183.9, and 132 mg/L, respectively, whereas those of DT were 481.9, 522.7, 685.0, and 406.0 mg/L, respectively (**Supplementary Table S6**). The inhibitory effects of DJ were dose-dependent, with complete suppression of S1 and S2 observed at 300 mg/L. In contrast, increasing DT concentrations did not markedly enhance its antifungal activity. Taken together, these results demonstrated that DJ exhibited stronger antifungal effects than DT, and both compounds could induce distinct changes in fungal colony morphology and mycelial structures, which might further affect fungal pathogenicity.

Furthermore, both DJ and DT were abundant in several bacterial supernatants (**Figure 5**), but the antifungal activity of each individual compound was weaker than that of corresponding bacterial supernatants. Therefore, we tested the antifungal activity of their mixtures at different ratios. When DJ and DT were mixed at a 7:3 ratio, the inhibitory effect against S1 was comparable to that of DJ alone (**Supplementary Figure S10B**). Moreover, the mixture at a 9:1 ratio inhibited S2 and S6 to a similar extent as DJ alone (**Supplementary Figure S10C, E**). However, the mixture exhibited a weaker inhibitory effect against S5 at all tested ratios compared to DJ alone. Importantly, the inhibitory effect of mixture increased with higher proportions of DJ. These results suggest that DJ and DT do not exhibit a clear synergistic interaction.

## 4 Discussion

*Parrotia subaequalis* is a Tertiary relict plant species with a highly restricted distribution in the mountainous regions of eastern China (Zhang et al., 2018). It is of great importance for both evolutionary ecology and ornamental horticulture (Yang et al., 2024; Zhang et al., 2025). However, in its native habitats, *P. subaequalis* is threatened by leaf spot disease and its incidence varied among these habitats (**Supplementary Figure S1** and **Supplementary Table S1**). Leaf disease not only reduces the ornamental value of its colorful leaves but also compromises photosynthetic efficiency and overall plant growth. Despite its ecological and horticultural importance, the causal agents of leaf spot disease in *P. subaequalis* remain largely unknown, and no effective biocontrol strategy has been developed. In this study, we provide the first evidence that *Alternaria* sp. S1, *Schizophyllum* sp. S2, *Diaporthe* sp. S5, and *Botryosphaeria* sp. S6 can cause leaf spot disease in *P. subaequalis* (**Figure 1**). Furthermore, we demonstrate that several bacterial endophytes isolated from *P. subaequalis* leaves can effectively suppress the growth of these fungal pathogens, and their metabolites also exhibit antifungal activities. It is interesting that leaves from HZ showed the lowest disease incidence, and 10 out of the 25 bacterial endophytes with antifungal activity were isolated from this location. These findings not only advance our understanding of interactions among host plants, fungal pathogens, and bacterial endophytes in a rare relict species but also highlight the potential of using bacterial endophytes and their bioactive metabolites for the development of sustainable disease management strategies to protect *P. subaequalis*.

The inoculation of *Alternaria* sp. S1, *Schizophyllum* sp. S2, *Diaporthe* sp. S5, and *Botryosphaeria* sp. S6 resulted in the development of obvious leaf spots on leaves of *P. subaequalis*, with S5 causing the most severe symptoms (**Figure 1B**). Previous studies have identified *Alternaria* species as pathogens causing leaf spot disease in various plants, such as apple (Harteveld et al., 2014) and grapevine (Yurchenko et al., 2024). Notably, *Alternaria* has a broad host range and thus poses a considerable threat to global food security (Fernandes et al., 2023). *Schizophyllum* species are typically associated with canker and rot diseases, predominantly affecting branches and trunks (Havenga et al., 2019). *Diaporthe* species are widely distributed and have been reported to cause leaf spot disease in grapevine (Fedele et al., 2024) and *Camellia sinensis* (Liu et al., 2024), as well as other diseases such as stem canker and fruit rot (Guarnaccia et al., 2018; Bai et al., 2023). Similarly, *Botryosphaeria* species are known to cause leaf spot disease in *Quercus dentata* (Liu et al., 2023), *Malania oleifera* (Pan et al., 2022), and *Populus deltoides* (Saini and Pandey, 2024). Additionally, they are recognized as some of the most widespread and destructive causal agents of canker and dieback in woody plants, infecting more than 20 plant genera worldwide (Marsberg et al., 2017). Collectively, these four fungal genera are not only capable of inducing leaf spot disease in *P. subaequalis* but also responsible for a broad spectrum of economically significant diseases affecting food and cash crops. Consequently, the development of effective management strategies is essential to mitigate the adverse impacts of these pathogens.

Although chemical fungicides are effective in suppressing a broad range of fungal pathogens, their extensive application often results in adverse environmental consequences, such as the reduction of soil microbiome diversity and stability (Zhang et al., 2024), and the contamination of water bodies through rainfall and irrigation (Fenta and Mekonnen, 2024), which can accelerate the emergence of fungicide-resistant pathogens. Compared with chemical approaches, biocontrol strategies are characterized by high specificity and ecological safety, thereby minimizing disturbances to the environmental microbiome (Gao et al., 2017b; Ayilara et al., 2023; Jia et al., 2023; Wang et al., 2024b). Among biocontrol resources, bacterial endophytes are recognized as natural reservoirs of beneficial microbes that can effectively protect host plants against pathogenic infections and associated damage (Wang et al., 2024b). In this study, a total of 206 bacterial endophytes were isolated from *P. subaequalis* leaves collected across six native habitats. Antagonistic assays demonstrated that bacterial endophytes belonging to *Bacillus*, *Stenotrophomonas*, *Rhizobium*, and *Peribacillus* could directly inhibit the growth of fungal pathogens (**Figure 3** and **Supplementary Figure S5**), with *Bacillus* strains showing the most pronounced antifungal activity. Specifically, *Bacillus* spp. YX7 and NB10 exhibited the strongest antagonistic effects against *Alternaria* sp. S1; *Bacillus* spp. NB14 and YX6 against *Schizophyllum* sp. S2; *Bacillus* sp. NB10 against *Diaporthe* sp. S5; and *Bacillus* sp. HZ4 against *Botryosphaeria* sp. S6. As well-known biocontrol agents, the antifungal activity of *Bacillus* are primarily mediated by the secretion of a wide spectrum of bioactive compounds, including peptides, lipopeptides, and volatile organic compounds (VOCs) (Gao et al., 2017b; Jinal et al., 2020). Additionally, *Bacillus* can produce hydrolytic enzymes such as chitinases and β-1,3-glucanases that degrade fungal cell walls, ultimately leading to pathogen cell death (Won et al., 2018). Here, the fermentation supernatants of *Bacillus* species significantly inhibited the growth of fungal pathogens (**Figure 4**), with the supernatant of *Bacillus* sp. HZ17 exhibiting the strongest antagonistic effects against *Alternaria* sp. S1; that of *Bacillus* sp. HZ25 against *Diaporthe* sp. S5; and those of *Bacillus* spp. YX7 and YX6 against *Botryosphaeria* sp. S6. Notably, the fermentation supernatants of all the tested *Bacillus* species significantly inhibited the growth of *Schizophyllum* sp. S2. Collectively, these findings highlight the strong biocontrol potential of bacterial endophytes and their metabolites in suppressing the fungal pathogens causing leaf spot disease in *P. subaequalis*. Based on these results, these effective bacterial endophytes could be combined to construct synthetic communities, which could then be applied for the comprehensive management of leaf spot disease in *P. subaequalis*.

The fermentation supernatants of some bacterial endophytes exhibited weaker antifungal activity than direct bacterial inoculation. It suggests that bacterial endophytes may sense fungal signals and synthesize specific metabolites that are absent in axenic fermentation, when they directly antagonize fungal pathogens (Carrión et al., 2019). Moreover, these strains may employ additional antagonistic mechanisms beyond the synthesis of soluble inhibitory compounds, such as competition with fungal pathogens for nutrients (Zhou et al., 2014), and the release of VOCs with antifungal activity (Wang et al., 2024b). Nevertheless, direct bacterial inoculation and fermentation supernatant treatment induced distinct fungal colony morphologies. For example, the fermentation supernatants of *Bacillus* sp. NB14, *Lysinibacillus* sp. HZ43, *Bacillus* sp. HZ30, *Neobacillus* sp. HZ27, *Bacillus* sp. HZ25, *Bacillus* sp. NB10, *Lysinibacillus* sp. HZ39, *Peribacillus* sp. YX8, *Stenotrophomonas* sp. NB7, and *Peribacillus* sp. HZ10 inhibited the melanin synthesis in *Alternaria* sp. S1 (**Figure 4A**), whereas their direct inoculation did not (**Figure 3A**). Melanin crosslinks with polysaccharides, enhancing the cell wall stability of *Alternaria* (Fernandes et al., 2021). Therefore, the impaired melanin synthesis in *Alternaria* sp. S1 may lead to a thinner cell wall with fewer infection pegs and more pores. Infection pegs are critical for *Alternaria* to penetrate plant tissues (Fernandes et al., 2023), while increased cell wall porosity may facilitate the entry of bacterial antifungal metabolites into fungal cells, ultimately suppress fungal growth and pathogenicity. These observations suggest that specific bacterial metabolites may contribute to antifungal activity through diverse mechanisms, thereby it is necessary to analyze the chemical composition of the bacterial fermentation supernatants.

Metabolite profiling was performed to identify compounds potentially responsible for the antifungal activities of bacterial fermentation supernatants. A total of 103 compounds absent in the LB medium were identified (**Figure 5**), among which dihydrojasmone (DJ) and δ-tridecalactone (DT) exhibited strong antifungal activity (**Figure 6**). Specifically, DJ significantly inhibited the growth of all four fungal pathogens, while DT significantly inhibited the growth of *Schizophyllum* sp. S2 and *Botryosphaeria* sp. S6. DJ, a jasmonic acid derivative, has been previously reported to suppress the growth of *Fusarium graminearum* and *Rhizoctonia solani* (Orsoni et al., 2020), and to inhibit pathogenic dermatophytes from *Microsporum* and *Trichophyton* by disrupting their plasma membrane function (Pinto et al., 2021). However, the antifungal activity of DT is reported here for the first time. Notably, DJ significantly inhibited melanin accumulation in *Alternaria* sp. S1 (**Supplementary Figure S8**), similar to the effects of bacterial fermentation supernatants. Microscopic examination further revealed that DJ induced mycelial wrinkling in S1, while DT caused similar wrinkling in both S1 and S2 (**Supplementary Figure S7**), which could impair mycelial extension and fungal pathogenicity. Furthermore, the fermentation supernatant of *Bacillus* sp. YX6 exhibited strong broad-spectrum antagonistic activity, with IR of 57.04% for S1, 99.68% for S2, 66.55% for S5, and 76.23% for S6 (**Figure 4**). Importantly, both DJ and DT were present at relatively high levels in this supernatant (**Figure 5B**); however, their individual antifungal effects were less pronounced than that of the whole fermentation supernatant. Therefore, the synergistic effects of DJ and DT were tested, but combinations at various ratios did not enhance antifungal activity relative to the individual compounds (**Supplementary Figure S10**). Considering that the fermentation supernatant of *Bacillus* sp. YX6 also contains other reported antifungal metabolites such as piperanine (Muharini et al., 2015), we speculate that the excellent antifungal activity of this strain may result from the combined action of multiple metabolites.

## 5 Conclusion

This study reported the fungal pathogens causing leaf spot disease in *Parrotia subaequalis* and highlighted the role of bacterial endophytes as natural reservoirs of antifungal agents. Combining pathogen identification, bacterial endophyte isolation, and function validation, we demonstrate that *Bacillus* species exhibited the most significant inhibitory effects among all the bacterial endophytes isolated from *P. subaequalis* leaves. Moreover, soluble compounds secreted by these strains, particularly dihydrojasmone (DJ) and δ-tridecalactone (DT), played a crucial role in suppressing fungal pathogens. These findings not only provide novel insights into host-pathogen-endophyte interactions in a rare relict species but also establish a scientific foundation for developing sustainable biocontrol strategies. Future studies should prioritize *in planta* validation to bridge laboratory discoveries with practical applications.

## Data Availability

The datasets presented in this study can be found in online repositories. The names of the repository/repositories and accession number(s) can be found below: https://www.ncbi.nlm.nih.gov/genbank/, PV233884-PV233890 https://www.ncbi.nlm.nih.gov/genbank/, PV266777-PV266982.

## Supplementary material

Supplementary Table 1 Geographic information of sampling sites and leaf spot disease incidence of *Parrotia subaequalis* across six native habitats.

Supplementary Table 2 Molecular and morphological characteristics of fungal pathogens isolated from *Parrotia subaequalis* leaves.

Supplementary Table 3 Taxonomic assignments of bacterial endophytes isolated from *Parrotia subaequalis* leaves based on 16S rRNA gene sequences.

Supplementary Table 4 Taxonomic assignments of the 25 antifungal strains based on *gyrB* gene sequences.

Supplementary Table 5 Specific metabolites detected in the filtered fermentation supernatants of 25 bacterial endophytes.

Supplementary Table 6 EC50 values of dihydrojasmone (DJ) and δ-tridecalactone (DT) against four fungal pathogens.

Supplementary Figure 1 Photographs of native habitats and *Parrotia subaequalis* plants in Xinyang, Henan Province.

Supplementary Figure 2 Phylogenetic tree of bacterial endophytes isolated from *Parrotia subaequalis* leaves.

Supplementary Figure 3 Schematic diagram illustrating the dual culture method used in the *in vitro* antifungal assays.

Supplementary Figure 4 Re-isolation of potential fungal pathogens.

Supplementary Figure 5 Heatmap showing inhibitory effects of 74 bacterial endophytes against four fungal pathogens.

Supplementary Figure 6 Partial least squares-discriminant analysis (PLS-DA) of filtered fermentation supernatants of 25 bacterial endophytes.

Supplementary Figure 7 Effects of dihydrojasmone (DJ) and δ-tridecalactone (DT) on fungal mycelial structure.

Supplementary Figure 8 Photographs of the fungal pathogen colonies treated with different concentrations of dihydrojasmone (DJ).

Supplementary Figure 9 Photographs of the fungal pathogen colony treated with different concentrations of δ-tridecalactone (DT).

Supplementary Figure 10 Inhibitory effects of different combination ratios of dihydrojasmone (DJ) and δ-tridecalactone (DT) on fungal pathogen growth.
