# Supplementary material for: Dihydrojasmone from antifungal bacterial endophytes as a biocontrol agent against leaf spot pathogens threatening the endangered Tertiary relict plant Parrotia subaequalis
Source: Front Plant Sci. 2025 Dec 4;16:1694888. doi: 10.3389/fpls.2025.1694888 (PMC12711764; doi:10.3389/fpls.2025.1694888)
Supplement: Supplementary Table 1 — Geographic information of sampling sites and leaf spot disease incidence of Parrotia subaequalis across six native habitats. [file DataSheet1.pdf]

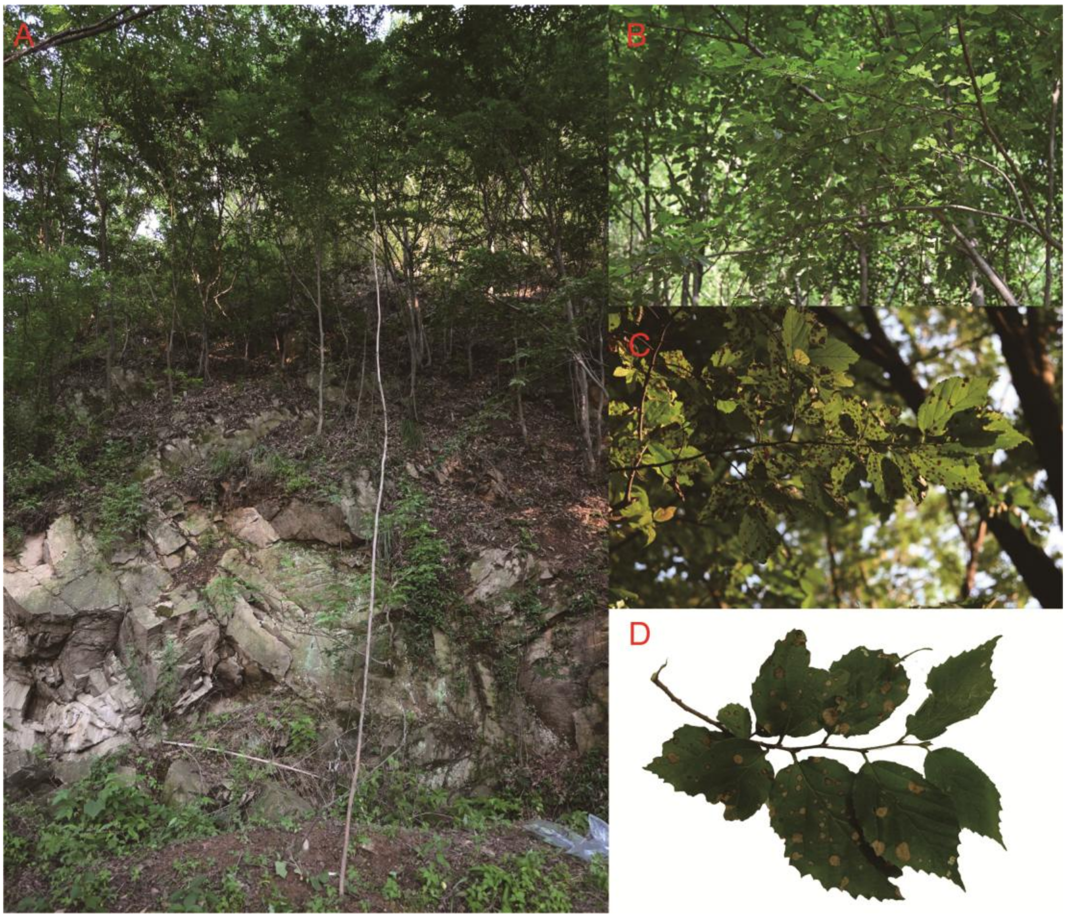

Supplementary Figure S1 Photographs of native habitats and *Parrotia subaequalis* plants in Xinyang, Henan Province. (A) Native habitat environment. **(B-C)** Leaves affected by leaf spot disease on the tree. **(D)** Detailed photograph of the diseased leaves.

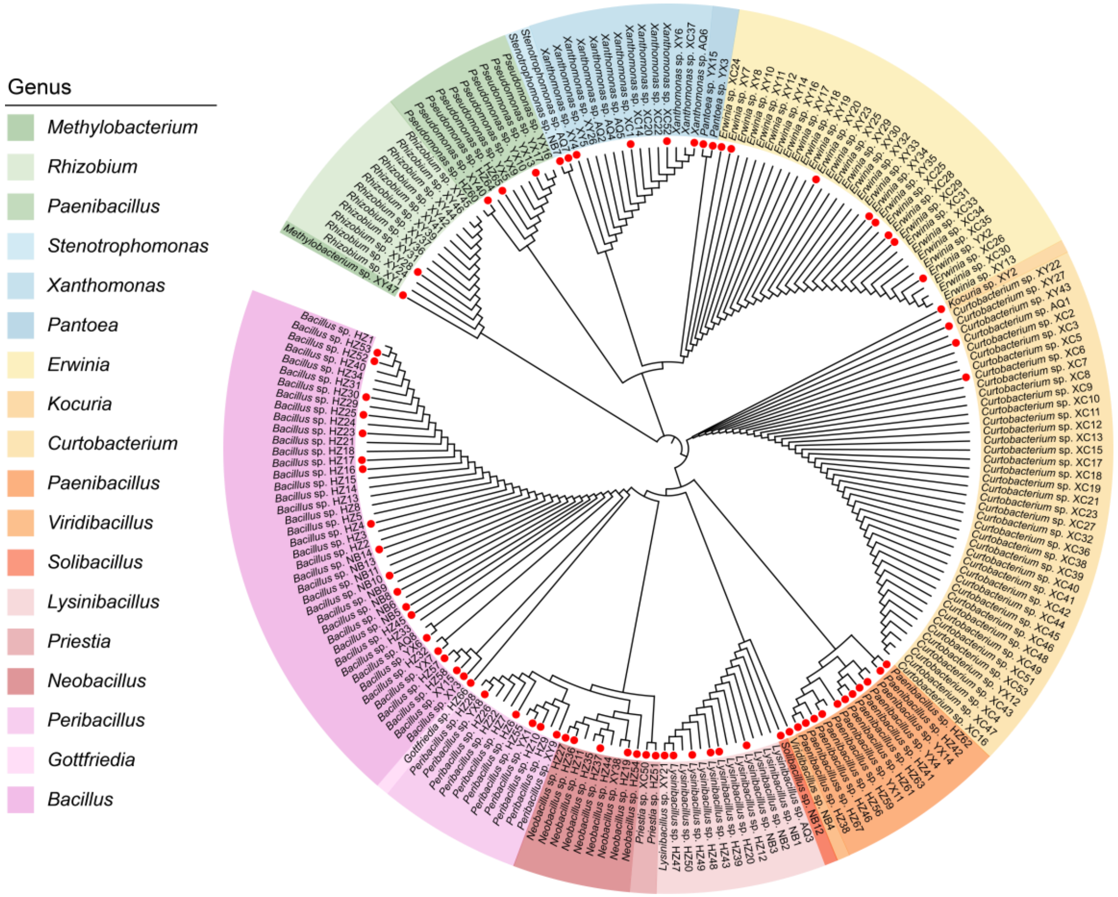

Supplementary Figure S2 Phylogenetic tree of bacterial endophytes isolated from *Parrotia subaequalis* leaves. Different bacterial genera are indicated in different colors. Strains marked with red dots are the ones used for preliminary antagonistic screening.

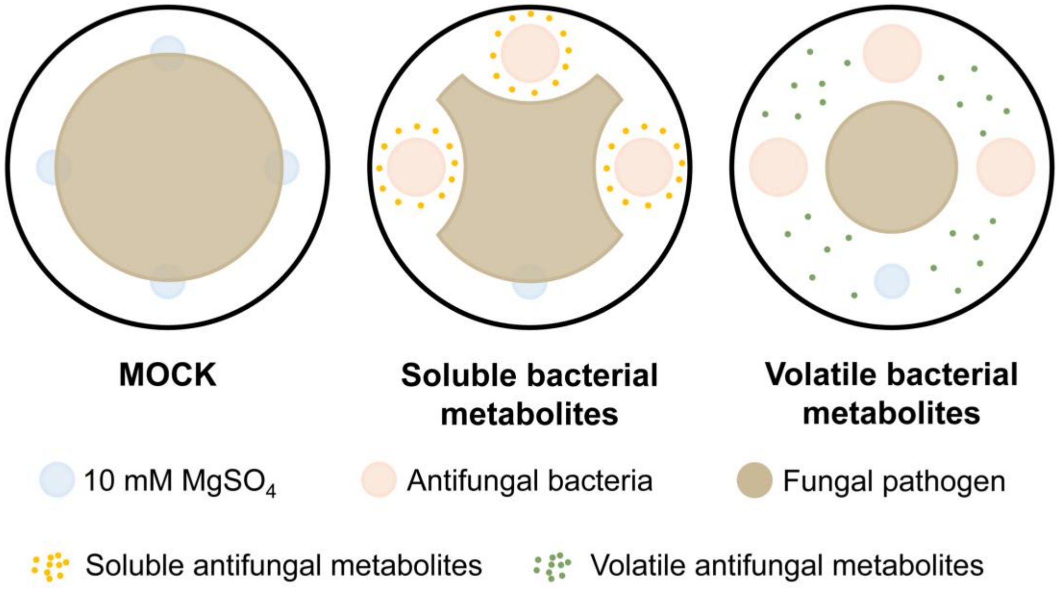

Supplementary Figure S3 Schematic diagram illustrating the dual culture method used in the *in vitro* antifungal assays.

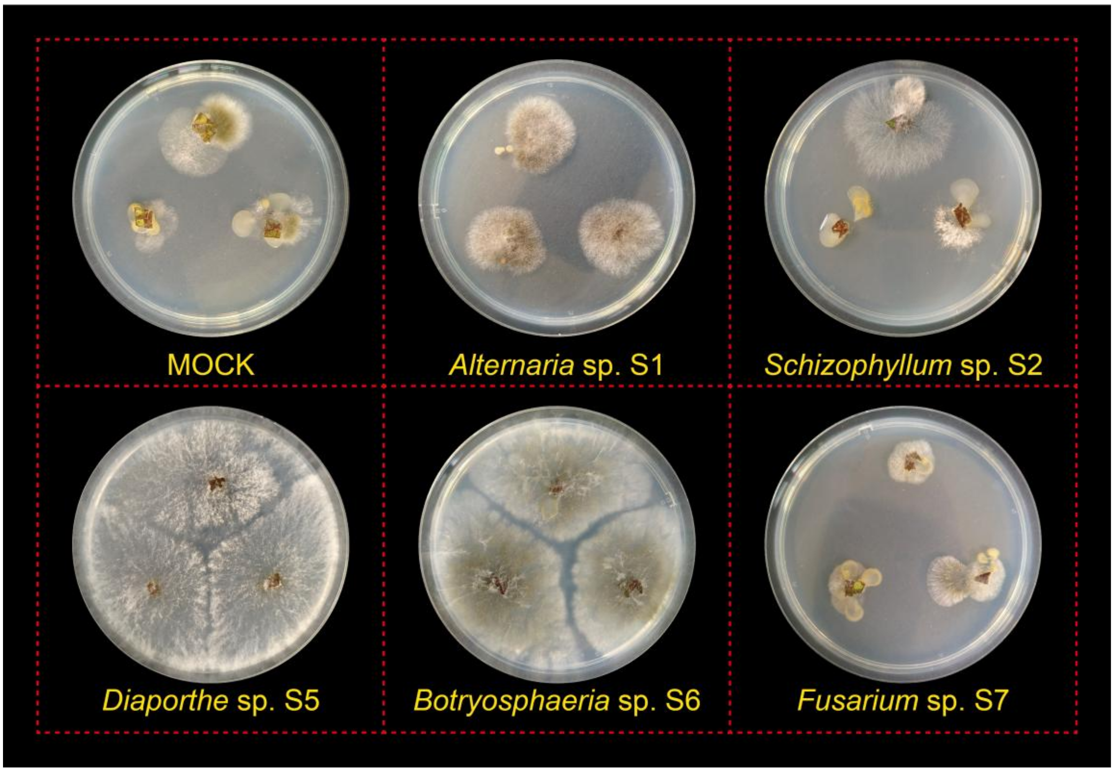

Supplementary Figure S4 Re-isolation of potential fungal pathogens. Fungal colonies of *Alternaria* sp. S1, *Schizophyllum* sp. S2, *Diaporthe* sp. S5, and *Botryosphaeria* sp. S6 re-isolated from inoculated *Parrotia subaequalis* leaves were the same as the original ones, indicating these fungal pathogens are responsible for leaf spot disease. In contrast, fungal colony of *Fusarium* sp. S7 is different from the original one.

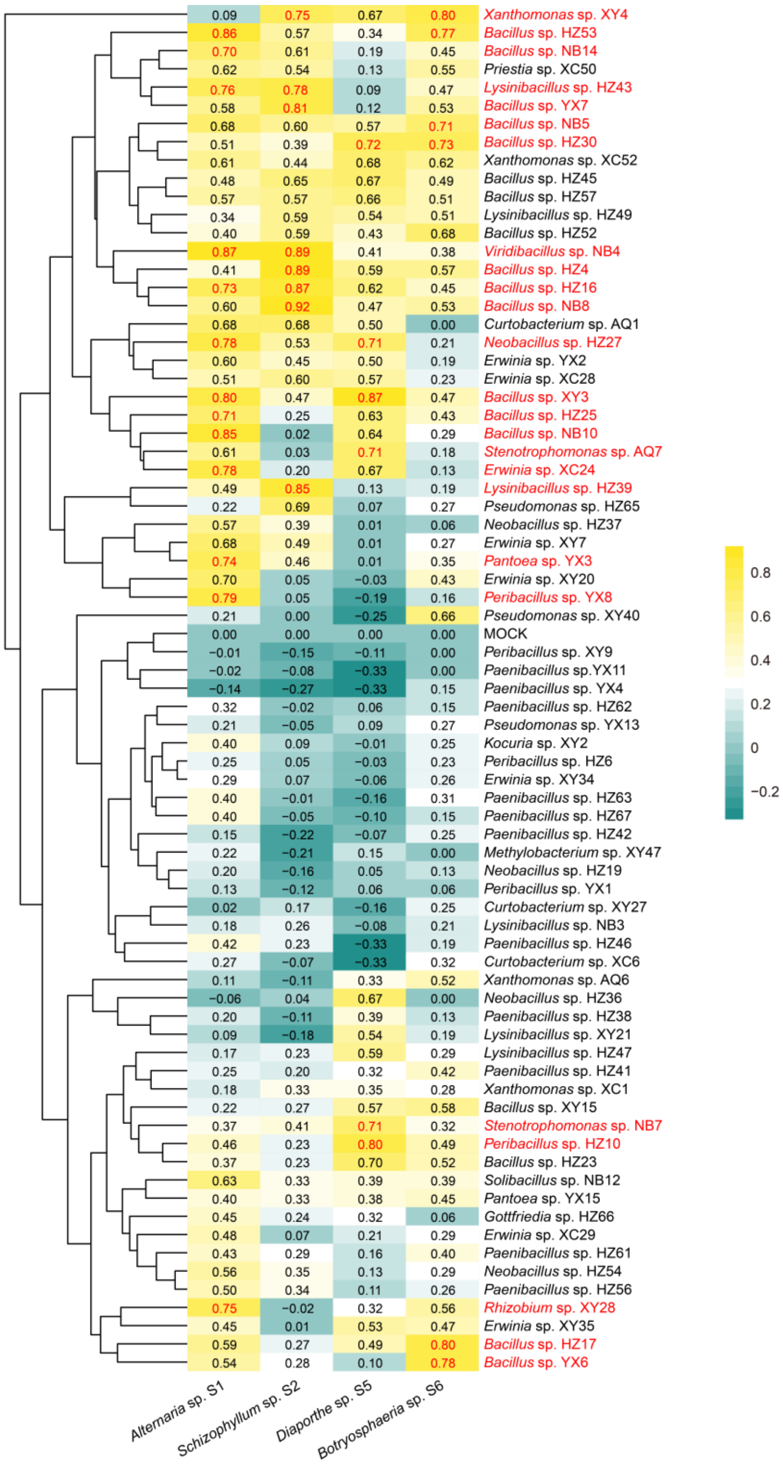

Supplementary Figure S5 Heatmap showing inhibitory effects of 74 bacterial endophytes against four fungal pathogens. Numbers show inhibition rates (IRs) of bacterial endophytes against fungal pathogens. Strains highlighted in red are selected for further antagonistic assays with an IR greater than 70%.

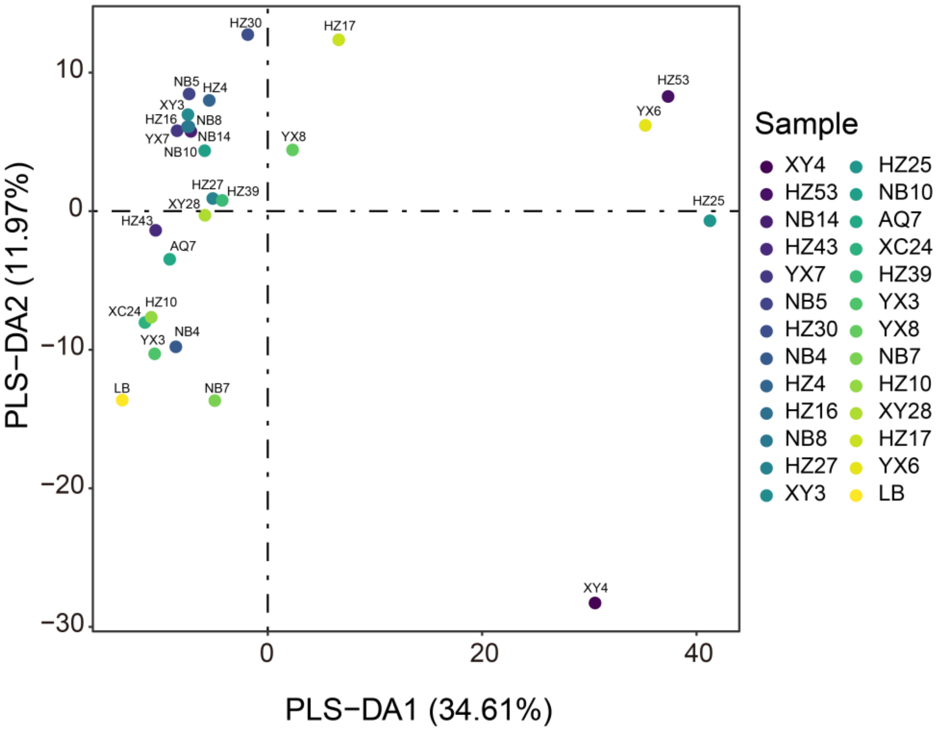

Supplementary Figure S6 Partial least squares-discriminant analysis (PLS-DA) of filtered fermentation supernatants of 25 bacterial endophytes.

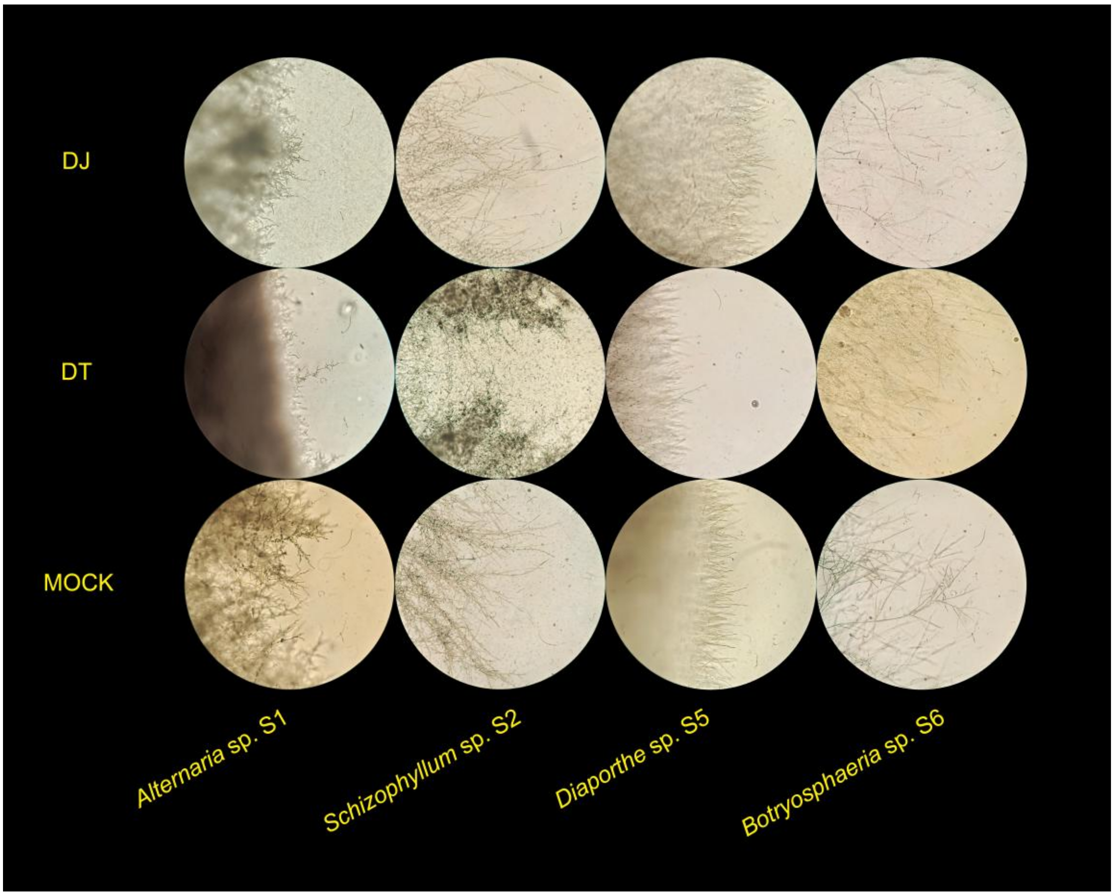

Supplementary Figure S7 Effects of dihydrojasmone (DJ) and δ-tridecalactone (DT) on fungal mycelial structure. The concentration of compounds was 200 mg/L, and fungal pathogens in MOCK group were treated with dimethyl sulfoxide.

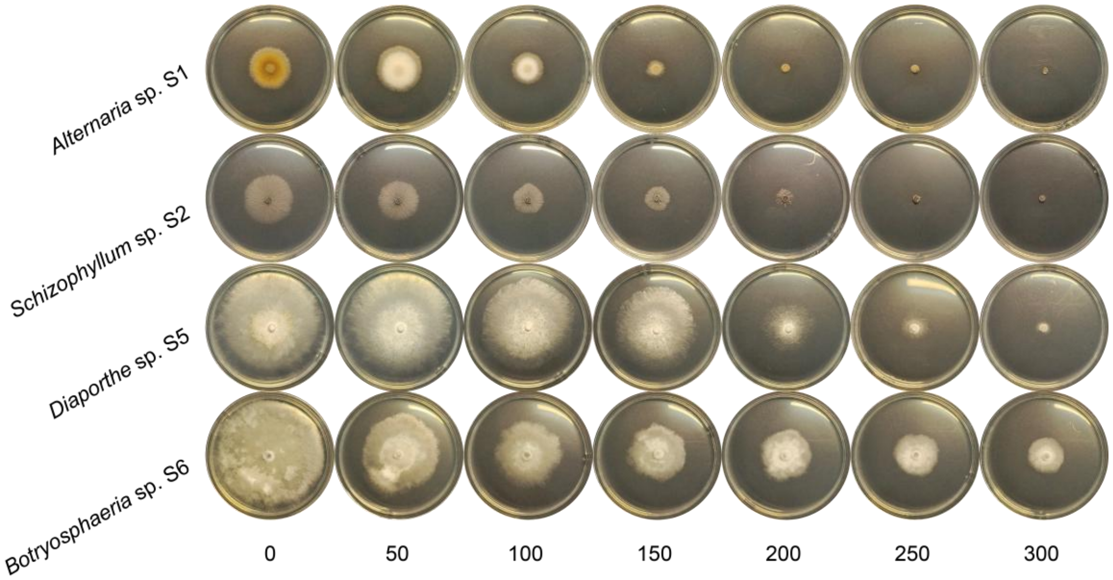

Supplementary Figure S8 Photographs of the fungal pathogen colonies treated with different concentrations of dihydrojasmone (DJ).

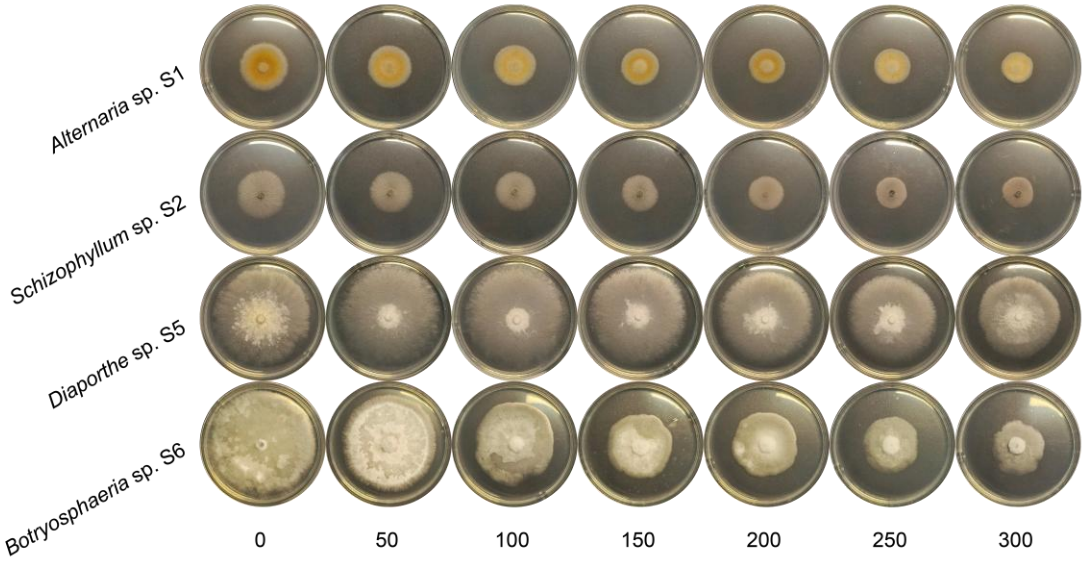

Supplementary Figure S9 Photographs of the fungal pathogen colonyies treated with different concentrations of δ-tridecalactone (DT).

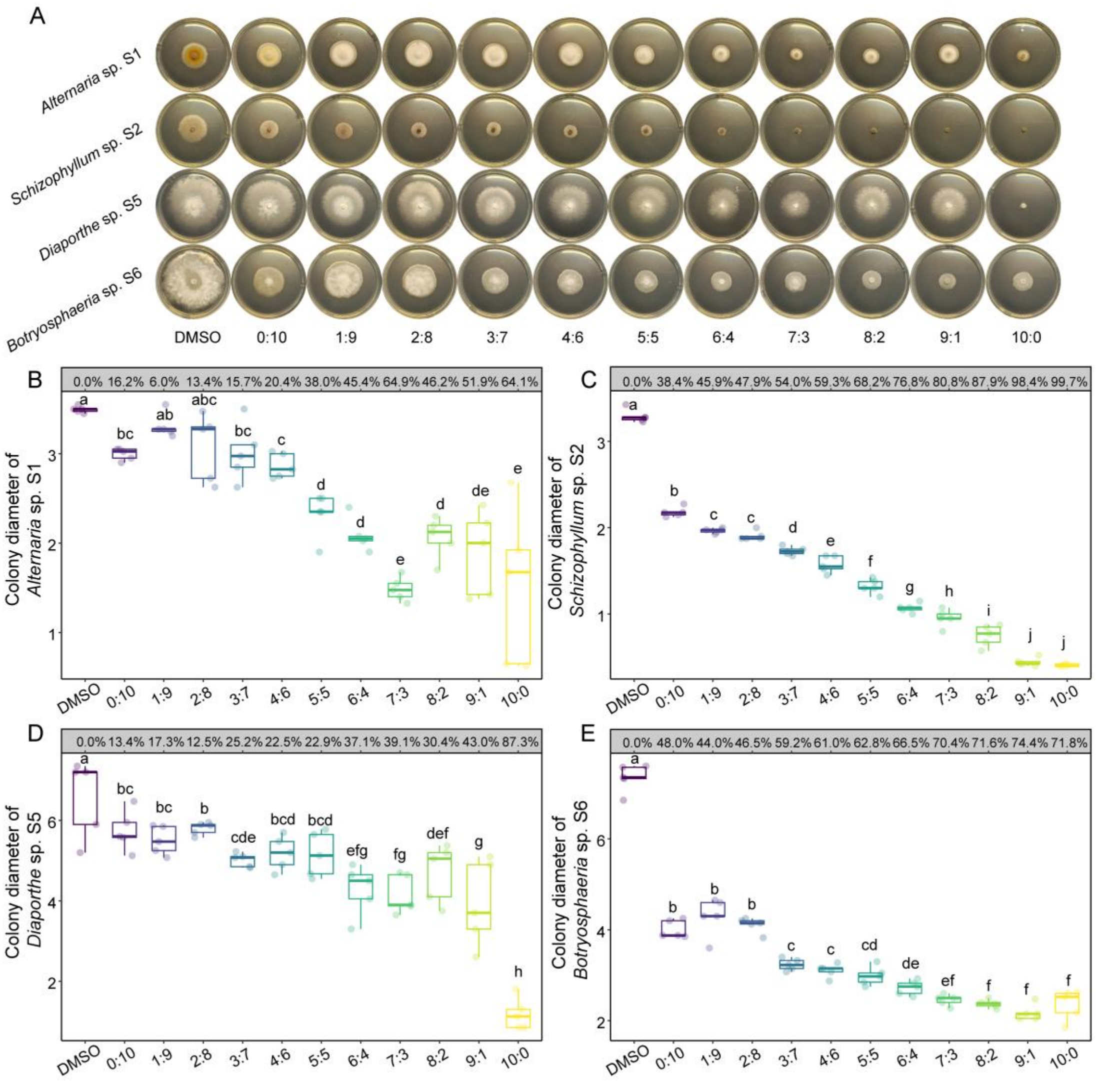

Supplementary Figure S10 Inhibitory effects of different combination ratios of dihydrojasmone (DJ) and δ-tridecalactone (DT) on fungal pathogen growth. (A) Representative images of fungal pathogens treated with DJ and DT combinations at different ratios. **(B-E)** Boxplots of colony diameters of *Alternaria* sp. S1 **(B)**, *Schizophyllum* sp. S2 **(C)**, *Diaporthe* sp. S5 **(D)**, and *Botryosphaeria* sp. S6 **(E)** treated by dimethyl sulfoxide (DMSO) or compound combinations at different ratios. The inhibition rates for each mixture are displayed in the grey boxes above the boxplots. The horizontal line inside each box represents the median; the top and bottom edges of each box represent the 75th and 25th quartiles, respectively; and the upper and lower whiskers extend to 1.5× the interquartile range from the top and bottom of the box,

respectively. Different letters represent significant differences among different ratios (One-way ANOVA with least significant difference test, $P < 0.05$, $N = 5$).
